# Supplementary material for: Environmental and Pathogenic Carbapenem Resistant Bacteria Isolated from a Wastewater Treatment Plant Harbour Distinct Antibiotic Resistance Mechanisms
Source: Antibiotics (Basel). 2021 Sep 16;10(9):1118. doi: 10.3390/antibiotics10091118 (PMC8472606; doi:10.3390/antibiotics10091118)
Supplement: Supplementary file 1 [file antibiotics-10-01118-s001.zip › antibiotics-1374910-supplementary.pdf]

Table S1. Concentrations of carbapenem resistant bacteria in the wastewater influent and discharged effluent after 24 h incubation at 30 °C or 42 °C. Values are expressed in colony forming units per millilitre.

		**Incubation at 30 °C**		**Incubation at 42 °C**	
		**CFU/mL influent**	**CFU/mL effluent**	**CFU/mL influent**	**CFU/mL effluent**
Biological sample 1	Plate 1	8.10E+04	5.60E+03	1.70E+03	1.14E+02
	Plate 2	9.00E+04	4.70E+03	2.20E+03	1.02E+02
	Plate 3	9.50E+04	4.90E+03	2.10E+03	1.25E+02
Biological sample 2	Plate 4	1.02E+05	3.50E+03	1.80E+03	1.41E+02
	Plate 5	1.05E+05	3.60E+03	1.40E+03	1.38E+02
	Plate 6	1.13E+05	4.30E+03	1.70E+03	1.38E+02
Biological sample 3	Plate 7	9.10E+04	7.10E+03	8.00E+02	1.04E+02
	Plate 8	9.50E+04	6.60E+03	3.00E+02	1.14E+02
	Plate 9	9.00E+04	8.00E+03	4.00E+02	1.08E+02
Mean ± standard deviation		9.58E+04 ± 9.58E+03	5.37E+03 ± 1.74E+03	1.38E+03 ± 7.82E+02	1.20E+02 ± 1.63E+01

Table S2. Species identification by 16S rRNA gene sequencing and screening of carbapenem resistance genes in the carbapenem resistant bacteria isolated from the wastewater influent and discharged effluent samples at 30 °C.

Sampling point	Species identification	Total isolated	Carbapenem resistance genes				
			*bla*KPC	*bla*OXA-48	*bla*NDM	*bla*IMP	*bla*VIM
Wastewater influent	*Aeromonas caviae*	7	3	-	-	-	-
	Aeromonas salmonicida	1	-	-	-	-	-
	Aeromonas veronii	7	3	-	-	-	-
	Pseudomonas entomophila	1	-	-	-	-	-
	Pseudomonas fluorescens	3	-	-	-	-	-
	Pseudomonas fragi	1	-	-	-	-	-
	Pseudomonas lundensis	1	-	-	-	-	-
	Pseudomonas migulae	1	-	-	-	-	-
	Pseudomonas psychrophila	1	-	-	-	-	-
	Pseudomonas putida	7	2	-	-	-	-
	Pseudomonas syringae	1	-	-	-	-	-
	Raoultella ornithinolytica	1	-	-	-	-	-
	Total	32	8	0	0	0	0
Discharged effluent	*Acinetobacter pittii*	1	-	-	-	-	-
	Aeromonas caviae	1	-	-	-	-	-
	Aeromonas veronii	8	-	-	-	-	-
	Chromobacterium rhizoryzae	3	-	-	-	-	-
	Pseudomonas entomophila	1	-	-	-	-	-
	Pseudomonas fluorescens	2	-	-	-	-	-
	Pseudomonas fragi	2	-	-	-	-	-
	Pseudomonas monteilii	1	-	-	-	-	-
	Pseudomonas psychrophila	1	-	-	-	-	-
	Pseudomonas putida	6	-	-	-	-	-
	Total	26	0	0	0	0	0

Table S3. Species identification by 16S rRNA gene sequencing and screening of carbapenem resistance genes in the carbapenem resistant bacteria isolated from the wastewater influent and discharged effluent samples at 42 °C.

Sampling point	Species identification	Total isolated	Carbapenem resistance genes				
			bla$_{KPC}$	*bla*$_{OXA-48}$	*bla*$_{NDM}$	*bla*$_{IMP}$	*bla*$_{VIM}$
Wastewater influent	*Acinetobacter baumannii*	6	4	-	-	-	2
	Acinetobacter pittii	5	3	1	-	-	1
	Citrobacter amalonaticus	1	1	-	-	-	-
	Citrobacter freundii	2	2	-	-	-	-
	Enterobacter asburiae	2	1	-	-	-	1
	Escherichia coli	6	4	-	-	-	2
	Klebsiella pasteurii	1	1	-	-	-	-
	Klebsiella pneumoniae	2	1	1	-	-	1
	Raoultella ornithinolytica	2	2	-	-	-	-
	Total	27	19	2	0	0	7
Discharged effluent	*Acinetobacter baumannii*	5	-	-	-	-	-
	Acinetobacter pittii	1	-	-	-	-	-
	Aeromonas veronii	2	-	-	-	-	-
	Citrobacter amalonaticus	1	1	-	-	-	-
	Citrobacter freundii	1	1	-	-	-	1
	Escherichia coli	4	4	-	-	-	-
	Klebsiella pneumoniae	8	8	-	-	-	-
	Total	22	14	0	0	0	1

Table S4. Taxonomic identification of the carbapenem resistant bacteria isolated from the discharged effluent samples at 30 °C using BLASTn, SpeciesFinder 2.0 and KmerFinder 3.2.

**BLASTn**	**SpeciesFinder 2.0**	**KmerFinder 3.2**
Pseudomonas fluorescens	*Pseudomonas fluorescens* *	*Pseudomonas fluorescens*
Pseudomonas fragi	*Pseudomonas fragi*	*Pseudomonas fragi*
Aeromonas caviae	*Aeromonas enteropelogenes*	*Aeromonas caviae*
Aeromonas veronii	*Aeromonas salmonicida*	*Aeromonas veronii*
Chromobacterium rhizoryzae	*Chromobacterium haemolyticum*	*Chromobacterium rhizoryzae*
Pseudomonas entomophila	*Pseudomonas putida* *	*Pseudomonas entomophila*
Pseudomonas monteilii	*Pseudomonas putida* *	*Pseudomonas monteilii*
Pseudomonas psychrophila	*Pseudomonas fragi*	*Pseudomonas fragi*
Pseudomonas putida	*Pseudomonas putida* *	*Pseudomonas* sp. URMO17WK12:I11
Acinetobacter pittii	*Acinetobacter* sp. 151 *	*Acinetobacter oleivorans*

* - Fail in the confidence of the result

Table S5. Taxonomic identification of the carbapenem resistant bacteria isolated from the discharged effluent samples at 42 °C using BLASTn, SpeciesFinder 2.0 and KmerFinder 3.2.

BLASTn	SpeciesFinder 2.0	KmerFinder 3.2
Aeromonas veronii	*Aeromonas veronii*	*Aeromonas veronii*
Klebsiella pneumoniae	*Klebsiella pneumoniae* *	*Klebsiella pneumoniae*
Acinetobacter baumannii	Acinetobacter sp. 148	*Acinetobacter baumannii*
Acinetobacter pittii	*Acinetobacter* sp. 159	*Acinetobacter pittii*
Escherichia coli	*Shigella sonnei*	*Escherichia coli*
Citrobacter amalonaticus	*Citrobacter amalonaticus*	*Citrobacter* sp. Y3
Citrobacter freundii	*Citrobacter freundii*	*Citrobacter portucalensis*

* - Fail in the confidence of the result

Table S6. Acquired AB resistance genes present in the whole genomes of the carbapenem resistant bacteria isolated from the discharged effluent samples at 30 °C.

Bacteria	β-lactams	Aminoglycosides	Fluoroquinolones	Macrolides	Phenicol	Rifampicin	Sulphonamides	Trimethoprim
Acinetobacter oleivorans	bla_{ADC-25}, $bla_{OXA-304}$	-	-	-	-	-	-	-
Aeromonas caviae	bla_{MOX-4}, bla_{MOX-6}	-	-	-	-	-	*sul*1	*dfr*A15
Aeromonas veronii	*amp*S, bla_{FOX-2}, bla_{MOX-3}, bla_{OXA-1}, bla_{OXA-10}, *cph*A1, *cph*A4, *cph*A7	*aac(3)-IId*, *aac(6′)-lb*, aac(6′)-*lb3*, *aac(6′)-lb-cr*, *aad*A1, *aph(3′)-Ia*, *aph(3′′)-lb*, *aph(6)-ld*	*aac(6′)-lb-cr*	*mph*A	*cat*B3	ARR-3	*sul*1, *sul*2	-
Chromobacterium rhizoryzae	-	-	-	-	-	-	-	-
Pseudomonas entomophila	-	-	-	-	-	-	-	-
Pseudomonas fluorescens	-	-	-	-	-	-	-	-
Pseudomonas fragi	-	-	-	-	-	-	-	-
Pseudomonas fragi	-	-	-	-	-	-	-	-
Pseudomonas monteilii	-	-	-	-	-	-	-	-
Pseudomonas sp. URMO17WK12:I11	-	-	-	-	-	-	-	-

Table S7. Acquired AB resistance genes present in the whole genomes of the carbapenem resistant bacteria isolated from the discharged effluent samples at 42 °C.

Bacteria	β-lactams	Aminoglycosides	Colistin	Fluoroquinolones	Fosfomycin	Macrolides	Phenicol	Sulphonamides	Tetracyclines	Trimethoprim
Acinetobacter baumannii	bla_{ADC25}, bla_{OXA-10}, bla_{OXA-24}, bla_{OXA-98}	*aad*A1, *aac(6′)-lb-cr*, *aac(6′)-lb3*	-	*aac(6′)-lb-cr*	-	*mph*E, *msr*E	-	-	-	-
Acinetobacter pittii	bla_{ADC-25}, $bla_{OXA-255}$, $bla_{OXA-506}$, $bla_{OXA-564}$	-	-	-	-	-	-	-	-	-
Aeromonas veronii	*amp*S, *cph*A4, *cph*A7	-	-	-	-	-	-	-	*tet*E	-
Citrobacter portucalensis	$bla_{CMY-129}$, bla_{KPC-2}, bla_{SHV-12}, bla_{TEM-1A}, bla_{TEM-1C}, bla_{TEM-40}, $bla_{TEM-150}$, $bla_{TEM-171}$, bla_{VIM-1}	*aad*A1, *aac(6′)-lb-cr*, *aac(6′)-lb3*, *aph(3′)-XV*	-	*aac(6′)-lb*-cr, *qnr*B6	*fos*A7	-	*cat*B2	*sul*1	*tet*A	*dfr*A1
Citrobacter sp. Y3	bla_{KPC-2}, bla_{OXA-1}, bla_{OXA-2}, bla_{TEM-1A}, bla_{TEM-1C}, bla_{TEM-40}, $bla_{TEM-150}$, $bla_{TEM-171}$	*aac(6′)-lb-cr*, *aac(6′)-lb3*	-	*aac(6′)-lb-cr*, *oqx*A, *oqx*B	-	-	*cat*B3	*sul*1	-	-
Escherichia coli	bla_{KPC-3}, bla_{OXA-9}, bla_{TEM-1A}	*aac(6′)-lb*, *aac(6′)-lb-cr*, *aad*A1, *aad*A2b, *ant(2′′)-la*, *aph(3′′)-lb*, *aph(6)-ld*	*mcr-9*	*aac(6′)-lb-cr*, *qnr*A1	-	-	-	*sul*1, *sul*2	-	*dfr*A14
Klebsiella pneumoniae	bla_{KPC-3}, bla_{OXA-9}, $bla_{SHV-101}$, bla_{TEM-1A}	*aad*A1, *aac(6′)-lb*, *aac(6′)-lb-cr*, *aph(3′′)-lb*, *aph(6)-ld*	-	*aac(6′)-lb*-cr, *oqx*A, *oqx*B	*fos*A	-	-	*sul*2	-	*dfr*A14

Table S8. Information about the sequence and amplicon size of the primers and probes used in each *TaqMan* qPCR reaction.

Gene	Primer/Probe	Sequence (5′ – 3′)	Amplicon (bp)
*bla*KPC	KPC Fw	GACGGAAAGCTTACAAAAACTGACA	259
	KPC Rv	CTTGTCATCCTTGTTAGGCG	
	KPC Probe	FAM-ACTGGGCAGTCGGAGACAAAACCGGA-BHQ1	
*bla*OXA-48	OXA-48 Fw	TTCGAATTTCGGCCACGG	204
	OXA-48 Rv	CATCAAGTTCAACCCAACCG	
	OXA-48 Probe	HEX-CCATGCTGACCGAAGCCAATGGTG-BHQ1	
*bla*NDM	NDM Fw	GGTTTGGCGATCTGGTTTTC	181
	NDM Rv	ATCCAGTTGAGGATCTGGGC	
	NDM Probe	FAM-CGGGGCAGTCGCTTCCAACGGTT-BHQ1	
*bla*IMP	IMP Fw	GGAATAGAGTGGCTTAAYTCTC	275
	IMP Rv	CAAGCTTCTATATTTGCGTCACC	
	IMP Probe	HEX-TTATCCAGGCCCGGGACACAC-BHQ1	
*bla*VIM	VIM Fw	GATGAGTTGCTTTTGATTGATACAGC	153
	VIM Rv	CGCCCGAAGGACATCAA	
	VIM Probe	ROX-ACGCACTTTCATGACGACCGCGTC-BHQ2	

Table S9. Information about the concentrations and volumes of mastermix, primers, probes, DNA templates and nuclease free water used in each *TaqMan* qPCR reaction.

***TaqMan* qPCR**	**Mix reaction**
TaqMan multiplex qPCR 1:<br>*bla*$_{KPC}$ and *bla*$_{OXA-48}$ genes	10 µL SensiFAST Probe No-ROX Kit (Bioline, London, UK)<br>400 nM *bla*$_{KPC}$ forward and reverse primers; 200 nM *bla*$_{OXA-48}$ forward and reverse primers<br>100 nM *bla*$_{KPC}$ and *bla*$_{OXA-48}$ *TaqMan* probes<br>50 ng *bla*$_{KPC}$ and *bla*$_{OXA-48}$ DNA templates<br>Nuclease free water (to make 20 µL)
TaqMan multiplex qPCR 2:<br>*bla*$_{NDM}$, *bla*$_{IMP}$ and *bla*$_{VIM}$ genes	10 µL SensiFAST Probe No-ROX Kit (Bioline, London, UK)<br>200 nM *bla*$_{NDM}$, *bla*$_{IMP}$ and *bla*$_{VIM}$ forward and reverse primers<br>100 nM *bla*NDM and *bla*IMP *TaqMan* probes; 10 nM *bla*$_{VIM}$ *TaqMan* probe<br>50 ng *bla*$_{NDM}$, *bla*$_{IMP}$ and *bla*$_{VIM}$ DNA templates<br>Nuclease free water (to make 20 µL)
